# Dichlorido(2-{[2-(piperazin-4-ium-1-yl)eth­yl]imino­meth­yl}phenolate)cadmium(II)

**DOI:** 10.1107/S1600536810032563

**Published:** 2010-08-18

**Authors:** Muhammad Saleh Salga, Hamid Khaledi, Hapipah Mohd Ali

**Affiliations:** a Department of Chemistry, University of Malaya, 50603 Kuala Lumpur, Malaysia

## Abstract

In the title compound, [CdCl_2_(C_13_H_19_N_3_O)], the Cd^II^ ion is penta­coordinated with the *N*,*N*,*O*-tridentate Schiff base 2-{[2-(piperazin-4-ium-1-yl)eth­yl]imino­meth­yl}phenolate ligand and two Cl atoms in a highly distorted square-pyramidal geometry; the piperazine ring adopts a chair conformation. In the crystal structure, adjacent mol­ecules are linked together *via* N—H⋯O and N—H⋯Cl hydrogen bonds, forming infinite layers parallel to the *ab* plane. The layers are further connected through C—H⋯Cl inter­actions into a three-dimensional network.

## Related literature

For related structures, see: Mukhopadhyay *et al.* (2003 ▶); Xu *et al.* (2008 ▶).
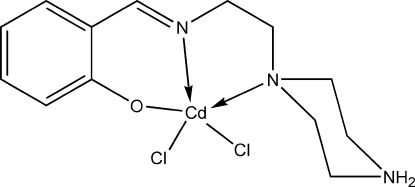


## Experimental

### 

#### Crystal data

[CdCl_2_(C_13_H_19_N_3_O)]
*M* *_r_* = 416.61
Orthorhombic, 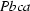

*a* = 14.7512 (16) Å
*b* = 13.1406 (15) Å
*c* = 16.6188 (19) Å
*V* = 3221.4 (6) Å^3^
*Z* = 8
Mo *K*α radiation
μ = 1.69 mm^−1^
*T* = 100 K
0.45 × 0.39 × 0.18 mm

#### Data collection

Bruker APEXII CCD diffractometer
Absorption correction: multi-scan (*SADABS*; Sheldrick, 1996 ▶) *T*_min_ = 0.518, *T*_max_ = 0.751
38841 measured reflections
3700 independent reflections
3409 reflections with *I* > 2σ(*I*)
*R*_int_ = 0.041

#### Refinement

*R*[*F* ^2^ > 2σ(*F* ^2^)] = 0.017
*wR*(*F* ^2^) = 0.045
*S* = 1.06
3700 reflections
187 parameters
2 restraints
H atoms treated by a mixture of independent and constrained refinement
Δρ_max_ = 0.44 e Å^−3^
Δρ_min_ = −0.28 e Å^−3^

### 

Data collection: *APEX2* (Bruker, 2007 ▶); cell refinement: *SAINT* (Bruker, 2007 ▶); data reduction: *SAINT*; program(s) used to solve structure: *SHELXS97* (Sheldrick, 2008 ▶); program(s) used to refine structure: *SHELXL97* (Sheldrick, 2008 ▶); molecular graphics: *X-SEED* (Barbour, 2001 ▶); software used to prepare material for publication: *SHELXL97* and *publCIF* (Westrip, 2010 ▶).

## Supplementary Material

Crystal structure: contains datablocks I, global. DOI: 10.1107/S1600536810032563/pv2318sup1.cif

Structure factors: contains datablocks I. DOI: 10.1107/S1600536810032563/pv2318Isup2.hkl

Additional supplementary materials:  crystallographic information; 3D view; checkCIF report

## Figures and Tables

**Table 1:** Hydrogen-bond geometry (Å, °)

*D*—H⋯*A*	*D*—H	H⋯*A*	*D*⋯*A*	*D*—H⋯*A*
N3—H3*A*⋯Cl1^i^	0.89 (2)	2.53 (2)	3.3108 (14)	146 (2)
N3—H3*A*⋯Cl2^ii^	0.89 (2)	2.78 (2)	3.2689 (14)	116 (2)
N3—H3*B*⋯O1^ii^	0.89 (2)	1.80 (2)	2.6743 (17)	167 (2)
C11—H11*B*⋯Cl2	0.99	2.70	3.6372 (16)	157
C13—H13*B*⋯Cl1	0.99	2.78	3.4302 (16)	124
C4—H4⋯Cl1^iii^	0.95	2.80	3.7165 (17)	161
C13—H13*A*⋯Cl1^i^	0.99	2.82	3.5851 (16)	134

Symmetry codes: (i) 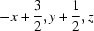; (ii) 
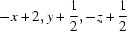
; (iii) 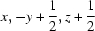.

**Fig. 1.**
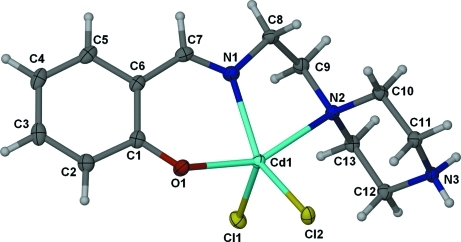
Displacement ellipsoid plot of the title compound at the 50% probability level. H atoms are drawn as spheres of arbitrary radius.

**Fig. 2.**
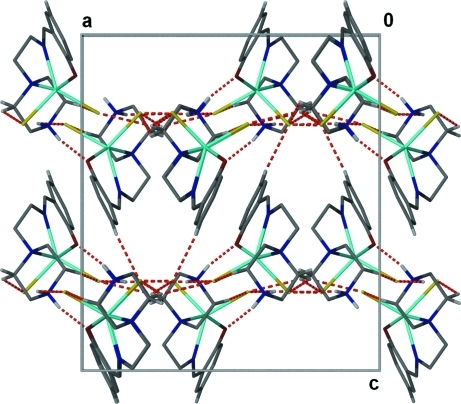
Unit-cell packing of the title compound, viewed down the b axis, showing the hydrogen-bonded polymeric network. H atoms not involved in hydrogen bonds have been omitted for clarity.

## supplementary crystallographic information

### Comment

The title compound is a cadmium (II) complex of the Schiff base ligand, 1-(2-salicylaldiminoethyl)piperazine. The piperazinyl arm of the ligand can, in principle, have both boat and chair conformations that makes the molecule to display ambidentate coordination behavior. The ligand has been shown to act as a tetradentate or tridentate chelate with nickel(II) ions, depending on the piperazine ring conformation (Mukhopadhyay *et al.*, 2003). In the title complex, the piperazine ring adopts the chair conformation and the ligand is bound to the metal ion in a NNO-tridentate fashion. The cadmium(II) atom is penta-coordinated by the Schiff base ligand and two chloride atoms in a highly distorted square planar geometry (index τ = 0.38). The piperzaine nitrogen atom, N3, which stays away from coordination, is protonated, implying the zwitterionic nature of the complex. In the crystal structure, intermolecular N—H···O, N—H···Cl and C—H···Cl hydrogen bonds connect the adjacent molecules into infinite three-dimensional network (Fig. 2). Morever, intramolecular C—H···Cl hydrogen bondings are observed. The crystal structure contains void spaces with the size of 199 Å^-3 ^(6.2% of the cell volume) within which there is no evidence for included solvent.

### Experimental

The Schiff base ligand was prepared following the procedure reported previously (Mukhopadhyay *et al.*, 2003). The cadmium (II) complex was synthesized by treatment of the ligand (0.233 g, 1 mmol) with cadmium (II) chloride (0.183 g, 1 mmol) in ethanol (20 ml). The mixture was stirred at room temperature for 10 min and then set aside for a few days whereupon the yellow crystals of the title compound were obtained.

### Refinement

The C-bound hydrogen atoms were placed at idealized positions (C—H = 0.95–0.99 Å) and were treated as riding on their parent atoms. The N-bound hydrogen atoms were located in a difference Fourier map and refined with distance restraint of N—H 0.88 (2) Å. The *U*_iso_(H) were allowed at 1.2*U*_eq_(C) or 1.5*U*_eq_(N). The final difference map was essentially featurless.

### Crystal data

**Table:**

[CdCl_2_(C_13_H_19_N_3_O)]	*F*(000) = 1664
*M**_r_* = 416.61	*D*_x_ = 1.718 Mg m^−^^3^
Orthorhombic, *P**b**c**a*	Mo *K*α radiation, λ = 0.71073 Å
Hall symbol: -P 2ac 2ab	Cell parameters from 9877 reflections
*a* = 14.7512 (16) Å	θ = 2.4–31.3°
*b* = 13.1406 (15) Å	µ = 1.69 mm^−^^1^
*c* = 16.6188 (19) Å	*T* = 100 K
*V* = 3221.4 (6) Å^3^	Block, yellow
*Z* = 8	0.45 × 0.39 × 0.18 mm

### Data collection

**Table:**

Bruker APEXII CCD diffractometer	3700 independent reflections
Radiation source: fine-focus sealed tube	3409 reflections with *I* > 2σ(*I*)
graphite	*R*_int_ = 0.041
φ and ω scans	θ_max_ = 27.5°, θ_min_ = 2.4°
Absorption correction: multi-scan (*SADABS*; Sheldrick, 1996)	*h* = −19→19
*T*_min_ = 0.518, *T*_max_ = 0.751	*k* = −17→17
38841 measured reflections	*l* = −21→21

### Refinement

**Table:**

Refinement on *F*^2^	Primary atom site location: structure-invariant direct methods
Least-squares matrix: full	Secondary atom site location: difference Fourier map
*R*[*F*^2^ > 2σ(*F*^2^)] = 0.017	Hydrogen site location: inferred from neighbouring sites
*wR*(*F*^2^) = 0.045	H atoms treated by a mixture of independent and constrained refinement
*S* = 1.06	*w* = 1/[σ^2^(*F*_o_^2^) + (0.0187*P*)^2^ + 2.1332*P*] where *P* = (*F*_o_^2^ + 2*F*_c_^2^)/3
3700 reflections	(Δ/σ)_max_ = 0.002
187 parameters	Δρ_max_ = 0.44 e Å^−^^3^
2 restraints	Δρ_min_ = −0.28 e Å^−^^3^

### Special details

**Table:**

Geometry. All e.s.d.'s (except the e.s.d. in the dihedral angle between two l.s. planes) are estimated using the full covariance matrix. The cell e.s.d.'s are taken into account individually in the estimation of e.s.d.'s in distances, angles and torsion angles; correlations between e.s.d.'s in cell parameters are only used when they are defined by crystal symmetry. An approximate (isotropic) treatment of cell e.s.d.'s is used for estimating e.s.d.'s involving l.s. planes.
Refinement. Refinement of *F*^2^ against ALL reflections. The weighted *R*-factor *wR* and goodness of fit *S* are based on *F*^2^, conventional *R*-factors *R* are based on *F*, with *F* set to zero for negative *F*^2^. The threshold expression of *F*^2^ > σ(*F*^2^) is used only for calculating *R*-factors(gt) *etc*. and is not relevant to the choice of reflections for refinement. *R*-factors based on *F*^2^ are statistically about twice as large as those based on *F*, and *R*- factors based on ALL data will be even larger.

### Fractional atomic coordinates and isotropic or equivalent isotropic displacement parameters (Å^2^)

**Table:**

	*x*	*y*	*z*	*U*_iso_*/*U*_eq_	
Cd1	0.915609 (7)	0.521115 (8)	0.335271 (6)	0.01335 (4)	
Cl1	0.80478 (3)	0.46142 (3)	0.23256 (2)	0.02000 (8)	
Cl2	1.05575 (2)	0.57074 (3)	0.26583 (2)	0.01803 (8)	
O1	0.98159 (7)	0.37354 (9)	0.37625 (7)	0.0192 (2)	
N1	0.87369 (9)	0.52146 (10)	0.46665 (8)	0.0162 (3)	
N2	0.83315 (8)	0.68754 (10)	0.35360 (8)	0.0139 (2)	
N3	0.88695 (9)	0.83051 (10)	0.22922 (8)	0.0149 (2)	
H3A	0.8533 (12)	0.8859 (13)	0.2359 (11)	0.022*	
H3B	0.9244 (12)	0.8437 (16)	0.1887 (11)	0.022*	
C1	0.95272 (10)	0.31114 (12)	0.43163 (9)	0.0169 (3)	
C2	0.96780 (11)	0.20498 (12)	0.42363 (10)	0.0195 (3)	
H2	0.9967	0.1802	0.3765	0.023*	
C3	0.94174 (11)	0.13662 (13)	0.48221 (10)	0.0220 (3)	
H3	0.9523	0.0660	0.4742	0.026*	
C4	0.90004 (11)	0.16929 (13)	0.55317 (10)	0.0213 (3)	
H4	0.8837	0.1221	0.5940	0.026*	
C5	0.88317 (10)	0.27220 (13)	0.56245 (9)	0.0194 (3)	
H5	0.8550	0.2953	0.6105	0.023*	
C6	0.90643 (10)	0.34411 (12)	0.50271 (9)	0.0167 (3)	
C7	0.87494 (10)	0.44702 (13)	0.51703 (9)	0.0172 (3)	
H7	0.8527	0.4611	0.5695	0.021*	
C8	0.83000 (10)	0.61616 (12)	0.49269 (9)	0.0178 (3)	
H8A	0.8766	0.6657	0.5100	0.021*	
H8B	0.7895	0.6024	0.5389	0.021*	
C9	0.77532 (10)	0.66006 (12)	0.42318 (9)	0.0172 (3)	
H9A	0.7296	0.6095	0.4058	0.021*	
H9B	0.7426	0.7214	0.4419	0.021*	
C10	0.88602 (10)	0.77994 (12)	0.37278 (9)	0.0164 (3)	
H10A	0.9254	0.7662	0.4198	0.020*	
H10B	0.8440	0.8357	0.3874	0.020*	
C11	0.94408 (10)	0.81319 (12)	0.30220 (9)	0.0158 (3)	
H11A	0.9765	0.8768	0.3162	0.019*	
H11B	0.9899	0.7602	0.2906	0.019*	
C12	0.82875 (10)	0.74064 (12)	0.21146 (9)	0.0166 (3)	
H12A	0.8674	0.6825	0.1954	0.020*	
H12B	0.7878	0.7567	0.1660	0.020*	
C13	0.77292 (10)	0.71146 (12)	0.28467 (9)	0.0158 (3)	
H13A	0.7321	0.7684	0.2992	0.019*	
H13B	0.7350	0.6514	0.2719	0.019*	

### Atomic displacement parameters (Å^2^)

**Table:**

	*U*^11^	*U*^22^	*U*^33^	*U*^12^	*U*^13^	*U*^23^
Cd1	0.01279 (7)	0.01314 (7)	0.01412 (7)	0.00111 (4)	0.00214 (4)	0.00129 (4)
Cl1	0.01838 (18)	0.01817 (18)	0.02346 (19)	−0.00298 (14)	−0.00124 (14)	−0.00369 (14)
Cl2	0.01453 (16)	0.01461 (17)	0.02495 (19)	−0.00003 (13)	0.00517 (14)	0.00176 (14)
O1	0.0201 (5)	0.0189 (5)	0.0186 (5)	0.0057 (4)	0.0045 (4)	0.0040 (4)
N1	0.0132 (6)	0.0189 (7)	0.0164 (6)	0.0015 (5)	0.0010 (5)	−0.0002 (5)
N2	0.0117 (6)	0.0146 (6)	0.0153 (6)	−0.0007 (5)	0.0005 (5)	0.0009 (5)
N3	0.0153 (6)	0.0127 (6)	0.0166 (6)	−0.0006 (5)	0.0005 (5)	0.0007 (5)
C1	0.0134 (7)	0.0180 (7)	0.0192 (7)	0.0003 (6)	−0.0030 (6)	0.0024 (6)
C2	0.0174 (7)	0.0190 (8)	0.0222 (8)	0.0013 (6)	−0.0020 (6)	0.0017 (6)
C3	0.0178 (7)	0.0175 (8)	0.0306 (9)	−0.0017 (6)	−0.0083 (6)	0.0054 (7)
C4	0.0167 (7)	0.0244 (8)	0.0229 (8)	−0.0052 (6)	−0.0065 (6)	0.0098 (7)
C5	0.0147 (7)	0.0273 (8)	0.0161 (7)	−0.0045 (6)	−0.0034 (6)	0.0050 (6)
C6	0.0143 (7)	0.0198 (8)	0.0158 (7)	−0.0019 (6)	−0.0024 (6)	0.0034 (6)
C7	0.0128 (7)	0.0241 (8)	0.0147 (7)	−0.0013 (6)	0.0000 (5)	0.0003 (6)
C8	0.0183 (7)	0.0195 (7)	0.0156 (7)	0.0017 (6)	0.0035 (6)	−0.0006 (6)
C9	0.0139 (7)	0.0179 (7)	0.0198 (7)	0.0011 (6)	0.0040 (6)	0.0009 (6)
C10	0.0153 (7)	0.0168 (7)	0.0172 (7)	−0.0021 (6)	0.0004 (6)	−0.0023 (6)
C11	0.0139 (7)	0.0155 (7)	0.0179 (7)	−0.0019 (6)	−0.0009 (6)	0.0003 (6)
C12	0.0164 (7)	0.0157 (7)	0.0177 (7)	−0.0023 (6)	−0.0025 (6)	−0.0004 (6)
C13	0.0124 (7)	0.0157 (7)	0.0192 (7)	−0.0007 (5)	−0.0020 (6)	0.0009 (6)

### Geometric parameters (Å, °)

**Table:**

Cd1—N1	2.2693 (13)	C4—C5	1.384 (2)
Cd1—O1	2.2740 (11)	C4—H4	0.9500
Cd1—Cl2	2.4557 (4)	C5—C6	1.413 (2)
Cd1—Cl1	2.4904 (4)	C5—H5	0.9500
Cd1—N2	2.5209 (13)	C6—C7	1.450 (2)
O1—C1	1.3042 (19)	C7—H7	0.9500
N1—C7	1.288 (2)	C8—C9	1.522 (2)
N1—C8	1.4667 (19)	C8—H8A	0.9900
N2—C10	1.4779 (19)	C8—H8B	0.9900
N2—C9	1.4816 (19)	C9—H9A	0.9900
N2—C13	1.4833 (19)	C9—H9B	0.9900
N3—C12	1.4897 (19)	C10—C11	1.517 (2)
N3—C11	1.4943 (19)	C10—H10A	0.9900
N3—H3A	0.889 (15)	C10—H10B	0.9900
N3—H3B	0.887 (15)	C11—H11A	0.9900
C1—C2	1.419 (2)	C11—H11B	0.9900
C1—C6	1.432 (2)	C12—C13	1.518 (2)
C2—C3	1.379 (2)	C12—H12A	0.9900
C2—H2	0.9500	C12—H12B	0.9900
C3—C4	1.398 (3)	C13—H13A	0.9900
C3—H3	0.9500	C13—H13B	0.9900
			
N1—Cd1—O1	80.22 (4)	C5—C6—C7	115.51 (14)
N1—Cd1—Cl2	132.92 (3)	C1—C6—C7	124.80 (14)
O1—Cd1—Cl2	90.39 (3)	N1—C7—C6	127.33 (14)
N1—Cd1—Cl1	118.77 (3)	N1—C7—H7	116.3
O1—Cd1—Cl1	102.57 (3)	C6—C7—H7	116.3
Cl2—Cd1—Cl1	108.309 (15)	N1—C8—C9	109.30 (12)
N1—Cd1—N2	75.55 (4)	N1—C8—H8A	109.8
O1—Cd1—N2	155.52 (4)	C9—C8—H8A	109.8
Cl2—Cd1—N2	103.45 (3)	N1—C8—H8B	109.8
Cl1—Cd1—N2	92.25 (3)	C9—C8—H8B	109.8
C1—O1—Cd1	127.43 (9)	H8A—C8—H8B	108.3
C7—N1—C8	117.32 (13)	N2—C9—C8	112.31 (12)
C7—N1—Cd1	128.33 (11)	N2—C9—H9A	109.1
C8—N1—Cd1	113.90 (9)	C8—C9—H9A	109.1
C10—N2—C9	109.61 (12)	N2—C9—H9B	109.1
C10—N2—C13	107.97 (12)	C8—C9—H9B	109.1
C9—N2—C13	108.04 (11)	H9A—C9—H9B	107.9
C10—N2—Cd1	118.96 (9)	N2—C10—C11	111.59 (12)
C9—N2—Cd1	99.25 (9)	N2—C10—H10A	109.3
C13—N2—Cd1	112.30 (9)	C11—C10—H10A	109.3
C12—N3—C11	111.41 (12)	N2—C10—H10B	109.3
C12—N3—H3A	110.6 (13)	C11—C10—H10B	109.3
C11—N3—H3A	109.8 (12)	H10A—C10—H10B	108.0
C12—N3—H3B	111.3 (14)	N3—C11—C10	110.68 (12)
C11—N3—H3B	107.1 (13)	N3—C11—H11A	109.5
H3A—N3—H3B	106.4 (19)	C10—C11—H11A	109.5
O1—C1—C2	120.06 (14)	N3—C11—H11B	109.5
O1—C1—C6	123.22 (14)	C10—C11—H11B	109.5
C2—C1—C6	116.72 (14)	H11A—C11—H11B	108.1
C3—C2—C1	122.04 (16)	N3—C12—C13	110.73 (12)
C3—C2—H2	119.0	N3—C12—H12A	109.5
C1—C2—H2	119.0	C13—C12—H12A	109.5
C2—C3—C4	121.21 (16)	N3—C12—H12B	109.5
C2—C3—H3	119.4	C13—C12—H12B	109.5
C4—C3—H3	119.4	H12A—C12—H12B	108.1
C5—C4—C3	118.25 (15)	N2—C13—C12	110.34 (12)
C5—C4—H4	120.9	N2—C13—H13A	109.6
C3—C4—H4	120.9	C12—C13—H13A	109.6
C4—C5—C6	122.12 (15)	N2—C13—H13B	109.6
C4—C5—H5	118.9	C12—C13—H13B	109.6
C6—C5—H5	118.9	H13A—C13—H13B	108.1
C5—C6—C1	119.56 (15)		

### Hydrogen-bond geometry (Å, °)

**Table:**

*D*—H···*A*	*D*—H	H···*A*	*D*···*A*	*D*—H···*A*
N3—H3A···Cl1^i^	0.89 (2)	2.53 (2)	3.3108 (14)	146.(2)
N3—H3A···Cl2^ii^	0.89 (2)	2.78 (2)	3.2689 (14)	116.(2)
N3—H3B···O1^ii^	0.89 (2)	1.80 (2)	2.6743 (17)	167.(2)
C11—H11B···Cl2	0.99	2.70	3.6372 (16)	157.
C13—H13B···Cl1	0.99	2.78	3.4302 (16)	124.
C4—H4···Cl1^iii^	0.95	2.80	3.7165 (17)	161.
C13—H13A···Cl1^i^	0.99	2.82	3.5851 (16)	134.

Symmetry codes: (i) −*x*+3/2, *y*+1/2, *z*; (ii) −*x*+2, *y*+1/2, −*z*+1/2; (iii) *x*, −*y*+1/2, *z*+1/2.
